# The genetic structure of *Turnip mosaic virus* population reveals the rapid expansion of a new emergent lineage in China

**DOI:** 10.1186/s12985-017-0832-3

**Published:** 2017-08-29

**Authors:** Xiangdong Li, Tiansheng Zhu, Xiao Yin, Chengling Zhang, Jia Chen, Yanping Tian, Jinliang Liu

**Affiliations:** 1 Laboratory of Plant Virology, Department of Plant Pathology, College of Plant Protection, Shandong Agricultural University, Tai’an, Shandong 271018 China; 2 College of Plant Science and Technology, Tarimu University, Alar, 843300 China; 3 College of Plant Sciences, Jilin University, Changchun, 130062 China; 4 Xuzhou Sweet Potato Research Center of Jiangsu, Suzhou, 221121 China

**Keywords:** *Turnip mosaic virus*, *Potyvirus*, Genetic structure, Population, China

## Abstract

**Background:**

*Turnip mosaic virus* (TuMV) is one of the most widespread and economically important virus infecting both crop and ornamental species of the family *Brassicaceae*. TuMV isolates can be classified to five phylogenetic lineages, basal-B, basal-BR, Asian-BR, world-B and Orchis.

**Results:**

To understand the genetic structure of TuMV from radish in China, the 3′-terminal genome of 90 TuMV isolates were determined and analyzed with other available Chinese isolates. The results showed that the Chinese TuMV isolates from radish formed three groups: Asian-BR, basal-BR and world-B. More than half of these isolates (52.54%) were clustered to basal-BR group, and could be further divided into three sub-groups. The TuMV basal-BR isolates in the sub-groups I and II were genetically homologous with Japanese ones, while those in sub-group III formed a distinct lineage. Sub-populations of TuMV basal-BR II and III were new emergent and in a state of expansion. The Chinese TuMV radish populations were under negative selection. Gene flow between TuMV populations from Tai’an, Weifang and Changchun was frequent.

**Conclusions:**

The genetic structure of *Turnip mosaic virus* population reveals the rapid expansion of a new emergent lineage in China.

## Background

Due to the error-prone nature of their RNA-dependent RNA polymerases, populations of plant RNA viruses are genetically heterogeneous and the genetic structure may change with time and environment [1, 2]. Studies of the genetic structure of viruses will provide information about the mechanisms and factors driving their evolution and help us to understand the molecular evolutionary history of viruses in relation to their dispersion and emergence of new epidemics [3].

*Turnip mosaic virus* (TuMV) is a species of the largest plant virus genus *Potyvirus* (family *Potyviridae*). TuMV has flexuous filamental particles of 700–750 nm long and can be transmitted by 40–50 species of aphids in a non-persistent manner [4, 5]. The TuMV genome consists of one single-stranded positive sense RNA molecule of approximately 9830 nucleotides (nt) and contains a large open reading frame (ORF) [6]. The genomic RNA is translated into a large polyprotein and a frame-shift protein. The large polyprotein are subsequently processed by the action of three viral-encoded proteinases (Pl, HC-Pro and NIa-Pro) into ten mature functional products [7, 8]. A frame-shift protein, P3N-PIPO, was reported to be involved in the pathogenesis and movement of TuMV [9, 10].

TuMV can infect plants of 300 species in 43 families, and is probably the most widespread and economically important virus infecting both crop and ornamental species of family *Brassicaceae* [11, 12]. In an extensive survey conducted in 28 countries, TuMV ranked second for crop yield losses [4]. TuMV is a highly variable and has many biological and serological strains [13–16]. According to its host range, TuMV isolates can be classified to two pathotypes, B (mainly infects plants of the genus *Brassica*) and BR (infects plants of both *Brassica* and *Raphanus*). The brassica-infecting TuMV isolates were categorized into four phylogenetic lineages, basal-B, basal-BR, Asian-BR and world-B, which correlated well with their differences in pathogenicity and geographical origin [17]. Most recently, a monophyletic sister lineage called ‘Orchis group’ was detected from wild orchids-infecting TuMV isolates, which are more likely the ancestor of TuMV [18]. As in other potyviruses [19], recombination is a frequent event in the evolution of TuMV. Intra- and inter-lineage recombinants are common in natural populations of TuMV and can be detected throughout the genome [6, 20–22]. The Chinese and Japanese TuMV isolates are part of the same population but are a discrete lineage [22, 23]. The gene flow between sub-populations of TuMV from Vietnam, Japan and China are frequent [20]. The basal-BR isolates have occurred over the whole Japanese islands and have evolved into four sub-lineages [23–25].

Previous studies showed that the TuMV isolates of China can be clustered to world-B and Asian-BR groups [10, 17, 24, 26, 27]. However, we have detected the existence of basal-BR isolates in China and reported the complete genomic sequences of two basal-BR isolates that represented two novel recombination patterns [6, 28]. Here, we studied the genetic structure of TuMV population in China and found that the basal-BR group of TuMV was expanding in China.

## Methods

### Virus samples, RNA extraction and sequencing

Leaf samples of radish from Heilongjiang, Jilin and Shandong provinces from 2005 to 2010 were collected. All the samples were biologically purified by three cycles of single lesion isolation in *Chenopodium amaranticolor a*nd propagated in *B. rapa*. Inoculated plants were maintained in a glasshouse at 25 °C.

Total RNAs were extracted from 100 mg TuMV-infected *B. rapa* leaves with the Invitrogen Trizol Kit following instructions of the manufacturer. The 3-terminus of TuMV (~1.1 kb) were amplified with RT-PCR using primers CP-F (5′-ATC TTC GAA GAT TAC GAA GA-3′) and CP-R (5′-CCT TGC TTC CTA TCA AAT G-3′) [29]. The fragments were cloned into pMD18-T vector (TaKaRa Biotechnology Dalian Co, Ltd) and sequenced by a ABI PRISM™ 377 DNA Sequencer. For each isolate, at least four clones from two separate PCR were sequenced. In case of any inconsistence, at least two more clones will be sequenced to obtain the consensus sequence.

### Recombination analysis

The sequences of 101 TuMV isolates and other 28 obtained from the GenBank database were subjected to recombination analyses using the software package RDP3, which assembled programs RDP [30], GENECONV [31], BOOTSCAN [32], MAXCHI [33], CHIMEARA [34] and SISCAN. The sequences were analyzed using the default settings for different detection programs and a Bonferroni-corrected *P*-value cut off of 0.05. The potential recombinants identified by the programs in RDP3 were re-checked using PHYLPRO [35]. The RDP, BOOTSCAN and SISCAN programs were based on phylogenetic methods, whereas GENECONV, MAXCHI and CHIMAERA programs were substitution methods, and the PHYLPRO program was a distance comparison method. Only those sequences with recombination supported by at least three programs or two kinds of methods and with *P*-value <1.0 × 10^−6^ were regarded as ‘clear’ recombinants; otherwise, they were called as ‘tentative’ recombinants [23, 25].

### Phylogenetic analysis of the TuMV population

Sequence alignments were performed using the CLUSTAL W program (Thompson et al., 1994). Phylogenetic tree of TuMV isolates excluding the recombinant ones was constructed using methods including Maximum Likelihood (ML) method that are packaged in the MEGA6.0 [36]. The CP gene of one *Narcissus yellow stripe virus* (NYSV) isolate was used as outgroup [37]. Bootstrap analysis was repeated 1000 times to evaluate the significance of the internal branches.

### Sequences diversity and population demography analysis

DnaSP version 5.10 was used to calculate the values of nucleotide diversity, Tajima’s D, Fu and Li’s D and F tests, haplotype diversity and nucleotide diversity [38–40]. Tajima’s D, Fu and Li’s D and F tests hypothesize that all mutations are selectively neutral. Tajima’s D test depends on the differences between the numbers of segregating sites and the average number of nucleotide differences. Fu and Li’s D test is related the differences between the number of singletons (mutations appearing only once among the sequences) and the total numbers of mutations. Fu and Li’s F test is based on the differences between the numbers of singletons and the average number of nucleotide differences among all pairs of sequences. Haplotype diversity refers to the frequency and number of haplotypes in the population. Nucleotide diversity estimates the average pairwise differences among sequences. The nucleotide diversities were calculated within and between groups. DnaSP version 5.10 [40] was also used to estimate the frequency distribution of the number of pairwise differences among all sequences. Mismatch distribution of all populations were estimated on all pairs of haplotypes present in a population [40]. Mismatch distribution analysis was based on 1000 simulated samples and used to evaluate whether a population had undergone sudden expansion or maintained constant size. In a recently expanded and still intact population, the majority of lineage coalescence events were expected to produce a smooth unimodal Poisson distribution around the time of expansion; otherwise, multimodal and ragged distribution was expected.

### Selection pressure, genetic differentiation and gene flow

The selection pressure was estimated by *d*_N_/*d*_S_ ratio, where *d*_N_ represented the average number of non-synonymous substitutions per non-synonymous site and *d*_S_ represented the average number of synonymous substitutions per synonymous site. The values of *d*_N_ and *d*_S_ were estimated separately by using the PBL method [41, 42] implemented in MEGA 6.0. When *d*_N_/*d*_S_ ratio = 1, it means that neutral selection had occurred; when *d*_N_/*d*_S_ < 1 or >1, it means that negative (purifying) or positive (diversifying) selection, respectively, had occurred. Genetic distances were calculated by Pamilo-Bianchi-Li (PBL) methods [41, 42].

Genetic differentiation between populations was examined by three permutation-based statistical tests, *K*s*, Z and Snn [43, 44]. *P* < 0.05 was considered as the criterion for rejecting the null hypothesis that there is no genetic differentiation between two subpopulations. The level of gene flow between populations was measured by estimating *F*_st_ (the inter-populational component of genetic variation or the standardized variance in allele frequencies across populations) and *Nm* using DnaSP 5.10 [40]. *F*_st_ ranges from 0 to 1 for undifferentiated to fully differentiated populations, respectively. Normally, an absolute value of *F*_st_ > 0.33 or *Nm* < 1 suggests infrequent gene flow, while absolute value of *F*_st_ < 0.33 or *Nm* > 1 suggests frequent gene flow.

## Results

### Identities between TuMV isolates from radish in China

We collected and biologically cloned 101 TuMV isolates from radish from 2004 to 2010 from Beijing, Hebei, Heilongjiang, Henan, Jilin and Shandong provinces, 94 of which are first reported here. The biological characteristics of all the isolates in *C. amaranticolor* in *B. rapa* showed necrotic lesions and similar mosaic respectively. A fragment of 1082 bp covering partial NIb gene (28 bp), complete CP gene (867 bp) and 3′-UTR (187 bp) was amplified from these isolates. The geographical origin of each isolate is listed in Table 1.

**Table 1:** Recombination sites and possible parent-like isolates

Isolate	Major parent	Minor parent	B-E	Software*	*P*-value	Z-value^#^
CHBJ1	WF1–04	WF7–06	15–1051	GB**S**3	2.07 × 10^−18^	9.7
CHBJ2	WF1–04	WF7–06	15–1051	GB**S**3	2.07 × 10^−18^	9.65
CHK16	WF-05	R4	17–387	BMC**S**3	1.028 × 10^−14^	9.43
CHK51	WF-05	R4	17–387	BMC**S**3	1.028 × 10^−14^	9.38
R	WF-05	R4	17–697	MC**S**3	1.028 × 10^−14^	8.92
R5	WF-05	R4	17–451	MC**S**3	1.028 × 10^−14^	10.6
WF2–06	WF1–04	WF7–06	15–1051	GB**S**3	2.07 × 10^−18^	10.2
WF3–06	WF1–04	WF7–06	10–1051	GB**S**3	2.07 × 10^−18^	10.2
WF3–07	WF1–04	WF7–06	10–1047	GB**S**3	2.07 × 10^−18^	10.2
WF8–08	WF1–04	WF7–06	10–1051	GB**S**3	2.07 × 10^−18^	10.1
WF10–07	TA15–08	WF3–08	91–786	MC**S**3	1.18 × 10^−8^	7.1
WFLB3	WF7–06	WF1–04	377–657	GB**S**3	2.07 × 10^−18^	10.6

The recombination crossover sites within CP-UTR of turnip mosaic virus were detected by the recombination detecting programs. The geographical origin of each isolate were showed in Fig. 2

*The programs supporting recombination event. R(RDP), G (Geneconv), B (Bootscan), M (Maxchi), C (Chimaera), S (Siscan) and 3 (3seq). The analysis was carried out with default settings for the different detection methods and a Bonferroni-corrected cutoff of 0.05. The program that has the greatest *P-*value was marked in bold. B-E represents the beginning and ending point of recombination

The cloned sequences excluding primers shared identities of 89.6% - 100% at nt level with other 28 Chinese TuMV radish sequences available in Genbank database. These 129 CP gene sequences showed identities of 88.2% - 100% at nt level and 91.3% - 100% at aa level. The identities of 44 TuMV isolates from Weifang were 89.6% -100% at nt level and 95.1% - 100% at aa level. Those of 37 isolates from Tai’an were 88.9% - 100% at nt level and 94.1–100% at aa level. The 12 Changchun isolates shared identities of 90.3% - 100% at nt level and 95.8% - 100% at aa level.

### Recombination analyses

Possible recombination events in the CP-UTR region of 129 radish isolates from China were detected with the program package RDP. Twelve of the sequences (11.4%) analyzed had ‘clear’ recombination. Among the recombinant isolates, WF0710 was the within-group recombinant of basal-BR isolates, with TA0815 as its major parent and WF0803 as the minor parent; others were between-group recombinants of Asian-BR and world-B isolates, most with WF-05 or WF1–04 of world-B as the major parent and WF7–06 or R4 of Asian-BR as minor parent; WFLB had WF7–06 as its major parent and WF1–04 as minor parent (Table 1).

**Fig. 1:**
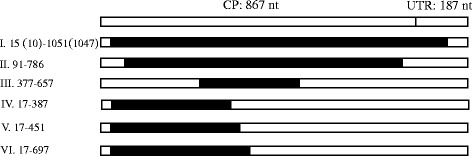
Recombination patterns in the CP-UTR region of TuMV from radish in China. Twelve recombinants were divided into 6 recombination patterns. I: CHBJ1, CHBJ2, WF2–06, WF3–06, WF8–08, WF3–07; II: WF10–07; III: WFLB3; IV: CHK16, CHK51; V: R5; VI: R

The recombination pattern can be classified into six types (Fig. 1). More than 50% recombinants belong to recombination pattern 1, with the recombination site located within UTR. WF0710 belonged to pattern II, WFLB3 to pattern III, CHK16 and CHK51 to pattern IV, R5 to pattern V and R to pattern VI (Fig. 1).

### Phylogenetic analyses

**Fig. 2:**
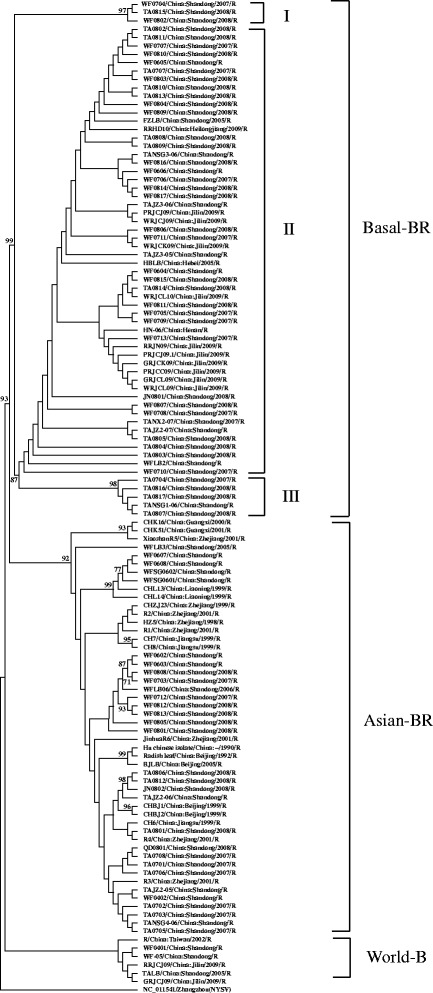
Maximum Likelihood tree of TuMV isolates from radish in China calculated from the CP -UTR sequences

Using ML method, a phylogenetic tree was constructed with the 118 CP-UTR sequences of TuMV (excluding the 11 between-group recombinants) from radish in China. These TuMV isolates were clustered to three lineages corresponding to world-B, Asian-BR and basal-BR (Fig. 2). The world-B lineage contained only six isolates (R, WF0401, WF-05, TALB, GRJCJ09 and RRJCJ09). The Asian-BR lineage consisted of 50 isolates. The Basal-BR lineage included 62 isolates which can be further divided into three sub-lineages. Sub-lineage Basal-BR I had three isolates (WF0704, WF0802 and TA0815), all of which were from Shandong province. Basal-BR II consisted of 54 isolates. Among which 41 were from Shandong, ten from Jilin, three from Henan, Heilongjiang and Hebei, respectively. Basal-BR III contained five isolates, all of which were found in Tai’an, Shandong province. The genetic distance values within groups ranged from 0.014 to 0.026, which were 4 to 5 times lower than those between groups (0.067 to 0.094) (Table 2). The genetic distance values between sub-groups of basal-BR were 0.033 to 0.041, which were higher than those within sub-groups but lower than those between groups.

**Table 2:** Estimates of genetic differentiation among sites (*F*_ST_) within each region

Gene	Parameter	WF between WF	TA between TA	WF between TA	WF between CC	TA between CC	CC between CC
CP	Ks(*P*-value)	3.16314 (1.0000)	3.12325 (1.0000)	3.14501 (0.1200)	3.01985 (0.0080**)	2.90505 (0.0120*)	2.41801 (1.0000)
	Z (*P*-value)	1935.22093 (1.0000)	1367.97222 (1.0000)	1618.33343 (0.2990)	740.62579 (0.0080**)	534.81963 (0.0060**)	143.22727 (1.0000)
	Snn (*P*-value)	0.08081 (1.0000)	0.12416 (1.0000)	0.66143 ** (0.0030)	0.88492 (0.0000***)	0.85305 (0.0000***)	0.10000 (1.0000)
	*F*_ST_	−0.02326	−0.02778	−0.00436	0.15467	0.15485	−0.09091
	*N*m	−11.00	−9.25	−57.56	1.37	1.36	−3.00
UTR	Ks*(*P*-value)	1.19299 (1.0000)	0.96193 (1.0000)	1.08658 (0.5460)	0.94143 (0.0570) ns	1.13222 (0.0420*)	0.88305 (0.9280) ns
	Z (*P*-value)	1844.07143 (1.0000)	1362.22222 (1.0000)	1581.52519 (0.4120)	552.71858 (0.0680) ns	723.74704 (0.0470*)	140.95455 (1.0000) ns
	Snn (*P*-value)	0.34906 (1.0000)	0.39139 (1.0000)	0.52678 (0.1430)	0.68796 (0.0230*)	0.69754 (0.0780) ns	0.31111 (1.0000) ns
	*F*_ST_	−0.02381	−0.02778	−0.01268	0.08893	0.11459	−0.09091
	*N*m	−10.75	−9.25	−19.96	2.56	1.83	−3.00

*WF* isolates from Weifang of Shandong province, *TA* isolates from Tai’an of Shandong province, *CC* isolates from Changchun of Jilin province

*ns* not significant; *, 0.01 < *P* < 0.05; **, 0.001 < *P* < 0.01; ***, *P* < 0.001. Determined using 1000 permutations

The phylogenetic tree constructed with the CP gene could also be divided into three groups corresponding to world-B, Asian-BR and basal-BR. The genetic distance values between groups ranged from 0.076 to 0.091, which were higher than those within groups (0.015 to 0.049). The genetic distance values between sub-groups of basal-BR were 0.032 to 0.049, which were remarkably higher than those within sub-groups (0.004 to 0.015) but lower than those between groups. Therefore, the classification of these TuMV isolates into three groups and basal-BR into three sub-groups was reliable.

**Fig. 3:**
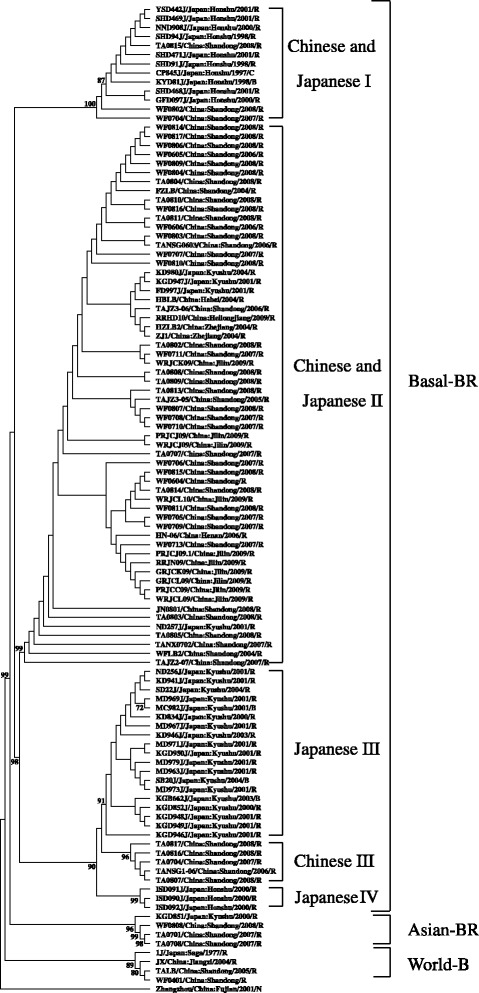
Phylogenetic trees were constructed by using the Maximum Likelihood method for CP gene nucleotide sequences of TuMV isolates of basal-BR collected from China and Japan. One *Narcissus yellow stripe virus* (NYSV) isolate (accession number: AJ311372) was used as outgroup. Isolates in the figure were listed by isolate name/location of origin/year of collection/original host (R is for *Raphanus*; B for *Brassica*; C for *Brassica* and *Raphanus*; N for *Not available*).

To further study the genetic structure of TuMV basal-BR sub-populations from China and Japan, we constructed phylogenetic trees with basal-BR isolates available from both countries using ML method (Fig. 3). These TuMV isolates were clustered into four lineages, corresponding to the ones reported by Tomitaka et al. [25]. Interestingly, the TuMV basal-BR isolates of sub-groups I and II from both China and Japan formed common clusters, which indicated sub-populations of basal-BR I and II from these two countries were genetically identical. However, those of sub-group III from China and Japan formed separate clusters, indicating that China and Japan had different sub-populations of basal-BR III. Sub-group IV consisted of isolates from Japan only. No Chinese TuMV isolate fell into this sub-group.

### Selective pressures acting on TuMV CP genes

To estimate the selection pressure acting on TuMV CP genes, we calculated the *d*_N_/*d*_S_ ratios for TuMV sub-populations of different collection regions using Pamilo-Bianchi-Li (PBL) method assembled in MEGA version 6.0 [36]. The *d*_N_ values for TuMV isolates from Weifang, Tai’an and Changchun were 0.005 ± 0.001, 0.006 ± 0.001 and 0.002 ± 0.001, respectively, which were less than the *d*_S_ values (0.070 ± 0.007, 0.098 ± 0.012 and 0.014 ± 0.006). Therefore, the values of the *d*_N_/*d*_S_ ratio for TuMV *cp* genes were <1, indicating that purifying (negative) selection was acting on TuMV *cp* genes. The nucleotide distances were 0.023 ± 0.002, 0.029 ± 0.003 and 0.005 ± 0.001, respectively, and showed no significant difference.

### Genetic differentiation and gene flow

Genetic differentiation and gene flow between and within populations was examined by five permutation-based statistical tests, Ks*, Z and Snn or *F*st and Nm. The results showed no genetic differentiation between or within TuMV sub-populations from Tai’an and Weifang in the CP genes or UTR (Table 2).

The absolute values of *F*_ST_ between or within TuMV populations of Tai’an, Weifang and Changchun were all below 0.33, indicating that the gene flow between or within TuMV populations of Tai’an and Weifang, and that with TuMV population of Changchun is most frequent; however, the gene flow between Changchun and Tai’an, and Changchun and Weifang is less frequent. The absolute values of *Nm* > 1 also support the conclusion on gene flow.

### Population dynamics

The Tajima’s *D*, Fu & Li’s D*, Fu & Li’s F* values for TuMV basal-BR II sub-population from Weifang and Tai’an of Shandong province were negative and the data is significant, which indicated that these sub-populations were in state of increasing (Table 3). Sub-populations of Asian-BR and basal-BRIII from Tai’an, Asian-BR from Weifang, and basal-BR from Changchun were also in a state of increasing, but the data was not significant (Table 3). Haplotype diversity, ranging from 0.890 to 1.000, had little difference between groups or sub-groups. The basal-BR II isolates from Tai’an had the lowest nucleotide diversity of 0.00322, while the Asian-BR isolates from Weifang had the highest one of 0.01159.

**Table 3:** Neutrality tests, haplotype and nucleotide diversity of *Turnip mosaic virus* sub-populations

Group		Haplotype diversity	Nucleotide diversity	Tajima’s D	Fu and Li’s D	Fu and Li’s F
Tai’an	basal-BR II	0.890 (0.073)	0.00322 (0.00062)	−2.19554**	−2.72246*	−2.72246**
	basal-BR III	1.000 (0.126)	0.00417 (0.00073)	−1.18441	−1.18441	−1.24511
	Asian-BR	0.939 (0.058)	0.00661 (0.00130)	−0.95449	−0.79711	−0.95432
Weifang	basal-BR II	0.960 (0.031)	0.00490 (0.00073)	−2.39975**	−3.45534 **	−3.66541**
	Asian-BR	0.987 (0.023)	0.01159 (0.00161)	−1.22279	−1.79120	−1.88684
Changchun	basal-BR	0.982 (0.046)	0.00478 (0.00095)	−1.63909	−2.06292	−2.21575

The sub-populations less than four isolates were not included

*: *P* < 0.05,**: *P* < 0.02

**Fig. 4:**
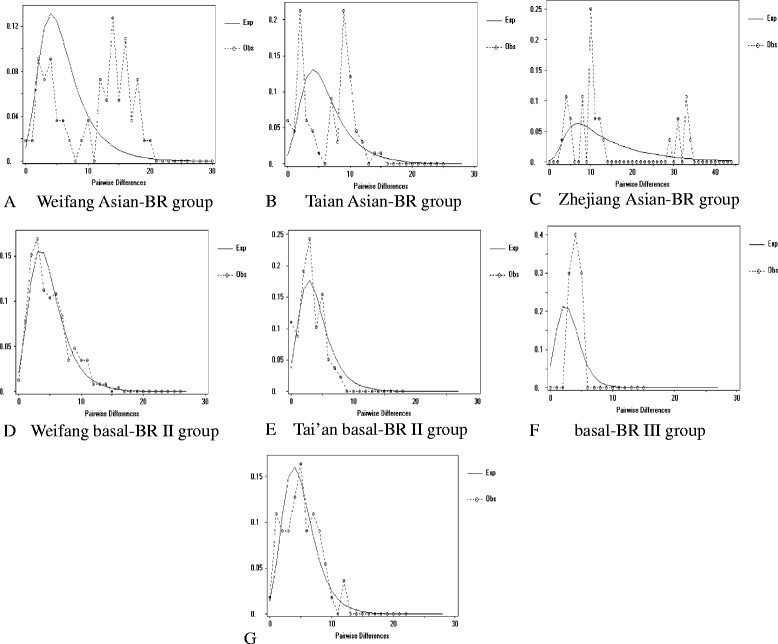
The frequency distribution of the number of pairwise nucleotide differences obtained from CP gene nucleotide sequences. **a** basal-BR II group of Weifang; **b** Asian-BR group of Weifang; **c** basal-BR II group of Tai’an, **d** basal-BR III group of Tai’an; **e** basal-BR II group of Tai’an, **f** basal-BR III group; **g** Changchun isolates of basal-BR group. Broken line represents the observed data and unbroken line represents the expected data. The sub-populations less than four isolates were not included

The mismatch distribution of TuMV CP gene and 3′-UTR for the basal-BR II isolates collected from Weifang, Tai’an, Changchun and basal-BR III were unimodual and smooth, and fit well with the expected model of sudden expansion, indicating that these sub-populations were new emergent (Fig. 4). The Asian-BR isolates from Weifang and Tai’an of Shandong province and Zhejiang were multiple-peaked, ragged, indicating that these sub-populations were long-existing ones (Fig. 4).

## Discussion

In this paper, we studied the molecular structure of TuMV population from China by analyzing the CP gene sequence of 129 TuMV isolates from radish and comparing them with 41 isolates of basal-BR group from Japan. Our results show that (1) about one-tenth of the TuMV isolates characterized are recombinants; (2) sub-population of basal-BR expands rapidly and accounting for more than one half of the isolates detected; (3) isolates of basal-BR in China evolve to three sub-groups, with sub-groups I and II genetically homologous with Japanese ones, while sub-group III a distinct lineage; (4) Sub-populations of TuMV basal-BR II and III are new emergent and in a state of expansion; (5) the TuMV population of China is under negative selection; (6) frequent gene flow is detected between TuMV sub-populations from Weifang, Taian and Changchun.

Recombination is important in virus evolution and has been detected in many potyvirus specie s [19, 20, 24, 45–48]. The percentages of recombinant isolates may accounting for ten to sixty-five of isolates studied [24, 47]. Intra- and inter-lineage recombination is very common in TuMV [6, 18, 22]. The hotspots of recombination sites of TuMV genome are located in the P1 and CI/6 K2/VPg region [21]. Ohshima and colleagues have detected 37 recombination patterns [6, 21]. Novel recombination patterns of TuMV are increasing [6, 18]. About 10% of the TuMV isolates characterized in this study experienced ‘clear’ recombination event. The percentage is a little lower than previous studies [18, 22]. The reasonable explanation might be that we just analyzed the CP-UTR region, where the crossover sites of TuMV are scarce [18, 22, 23]. If longer sequences or the whole genome is included, there would be more recombination events detected.

The *d*_N_/*d*_S_ ratios are often used to estimate the selection pressure under which viral gene (s) suffered [10, 49]. Positive selection (*d*_N_/*d*_S_ > 1) may endow the virus more fitness to adapt a new host or environment. However, rapid divergence driven by positive selection has been rarely demonstrated [50]. Like the case of most virus genes, our results show that negative (purifying) selection dominates the evolution of TuMV CP genes. If selection pressure on single residue is estimated, amino acids under positive selection may be sorted out [49].

Basal-BR is a new emergent in east Asia and has been detected in Japan and China [6, 17, 23, 28]. So far, there has been no basal-BR isolates reported in Vietnam [20]. After its first detection in 2005 [28], the population of basal-BR isolates increased rapidly in China and showed characteristics of founder effect. As reported in this research, Basal-BR isolates were detected from samples from Hebei, Henan, Jilin and Shandong provinces, and accounted to more than half of the isolates from Shandong and Jilin provinces. The Chinese basal-BR isolates have evolved to three sub-groups. Among the 48 basal-BR isolates from Weifang and Tai’an of Shandong province, 40 belonged to sub-group II, which represents the prevalent cluster in those areas. Basal-BR III was detected after 2006 and only found in Tai’an of Shandong province. What’s more interesting, sub-groups of basal-BR I and II are genetically homologous to those of Japanese isolates, while sub-groups of basal-BR III from China and Japan are genetically distinct and form separate clusters, indicating that China and Japan had different sub-populations. Another difference is that the prevalent subgroup of Basal-BR is II in China but III in Japan.

The gene flow between TuMV isolates of basal-BR II and III from Weifang, Tai’an and Changchun is frequent. But TuMV is transmitted by aphid in a non-persistent manner and there is no evidence of seed transmission reported [4, 5]. It remains unknown how TuMV isolates, especially the new emergent, spread to other places [18, 23, 25]. But TuMV isolates of basal-BR II are prevalent and expanding rapidly in Weifang and Tai’an of Shandong and Changchun of Jilin. Therefore, a program should be launched to evaluate the resistance of commercial available cultivars of cruciferous crops to TuMV isolates, particularly basal-BR II.

## Conclusions

Genetic structure of TuMV population in China reveals that the basal-BR group of TuMV was expanding, which was a new emergent lineage in China.

## Abbreviations

CP: Coat protein
NYSV: *Narcissus yellow stripe virus*
ORF: Open reading frame
PBL: Pamilo-Bianchi-Li
PIPO: Pretty Interesting Potyviridae
RdRP: RNA-dependent RNA polymerase
TuMV: *Turnip mosaic virus*
